# Social Media Posts from Friends during Late Adolescence as Predictors of Young Adult Physical Health

**DOI:** 10.1007/s10964-024-01945-4

**Published:** 2024-01-29

**Authors:** David E. Szwedo, Alida A. Davis, Caroline Fowler, Amori Yee Mikami, Joseph P. Allen

**Affiliations:** 1 Department of Graduate Psychology, James Madison University, 70 Alumnae Dr./MSC 7401, Harrisonburg, VA 22807, USA; 2 Department of Psychology, University of Virginia, P.O Box 400400, Charlottesville, VA 22904, USA; 3 Department of Psychology, University of British Columbia, 2136 West Mall, Vancouver, BC V6T 1Z4, Canada

**Keywords:** Health, Social media, Facebook, Peer relationships, Social integration, Online

## Abstract

Although an increasing body of literature has linked social experiences to physical health, research has yet to consider how specific aspects of social experiences taking place on social media during late adolescence may predict future physical health outcomes. This study thus examined qualities of social media posts received from peers at age 21 as predictors of participants’ physical health (e.g., Interleukin-6 (inflammation), sleep problems, problems with physical functioning, and BMI) at age 28. Participants included 138 youth (59 men and 79 women); 57% of participants identified as White, 30% as Black/African American, and 13% as from other or mixed racial/ethnic groups. Posts from friends and participants at age 21 characterized by social ties predicted lower levels of future physical health problems, whereas socially inappropriate “faux pas” posts that deviated from peer norms by friends predicted higher levels of physical health problems at age 28. These associations were found after accounting for factors typically associated with physical health outcomes, including participants’ baseline social competence, internalizing and externalizing symptoms, alcohol use, observed physical attractiveness, and history of prior hospitalizations. The results of this study suggest the importance of both achieving social integration with peers online and adhering to peer norms in the online domain as key predictors of future physical health.

## Introduction

Decades of research indicate that social integration – the extent to which one has meaningful, positive social ties and with many other people or a large peer group – may “get under the skin” to shape trajectories of health and disease (Holt-Lunstad et al., 2010). For example, individuals with a greater number of friendships may have improved general health outcomes (Ho, 2016), while those who lack social support may experience elevated rates of cardiovascular disease (Czajkowski et al., 2022). But the rise of social media has ushered in a new era of expanded social experiences, the consequences of which have yet to be fully explored (Nesi et al., 2018a). Scholars have theorized that social experiences that take place online might not simply mirror those that take place offline; rather, they might be “transformed”— rendered uniquely intense and impactful by the social media context (Nesi et al., 2018b). That is, the perpetual accessibility, permanence, and publicity afforded by social media platforms may amplify the psychological and physiological sequelae of the social experiences that unfold upon them. It is critical to understand the specific ways in which online social integration may predict or shape physical health outcomes, particularly for youth, among whom the use of social media is now near ubiquitous (Auxier & Anderson, 2021). Therefore, the aim of the present study is to examine how key aspects of social integration represented in posts on late adolescents’ social media may be predictors of their subsequent health outcomes as young adults.

Studies that have been conducted “offline” suggest that social integration is a particularly potent predictor of physical wellbeing for people of all ages. Strong social ties, one aspect of social integration, is associated with reduced inflammation (Uchino et al., 2018), improved sleep (de Grey et al., 2018), more active antiviral responses (Leschak & Eisenberger, 2019), and lower rates of all-cause mortality (Holt-Lundstad et al., 2010). Conversely, the health risks posed by lacking social ties are comparable to the effects of smoking a pack of cigarettes a day (Holt-Lundstad et al., 2010). Another aspect of social integration is indicated by the individual fitting in with the social norms of the broader peer group. In line with findings on the benefits of strong social ties, an emerging literature also suggests that fitting in with the peer group may predict physical health outcomes (Allen et al., 2015). Adolescents and adults who adhere to social norms have been found to report better global health outcomes up to a decade later (Ferguson et al., 2020), and individuals who value maintaining social harmony have been found to have lower rates of hypertension (Merz et al., 2016). In a study of patients with ulcerative colitis, those who demonstrated an above-average level of social conformity reported experiencing less symptom severity (Boye et al., 2008). Finally, another study found that teens who maintained unusually time-consuming romantic relationships in adolescence—in other words, those teens whose relationships were out of step with developmental norms— experienced heightened blood pressure 14 years later (Allen et al., 2021).

The achievement of social integration may be so influential on physical health because of the evolutionary necessity of group acceptance and cooperation for survival. Social baseline theory (SBT; Beckes & Coan, 2011) posits that the human brain innately expects access to supportive others who can assist in necessary survival goals. In the actual or anticipated absence of enough supportive others, the brain perceives a heightened level of threat and acts accordingly, e.g., increasing vigilance and preparing the body for conservation of resources (Gross & Medina-DeVilliers, 2020). Sustained neural perceptions of threat have been associated with metabolic shifts such as increased basal glucose levels and alterations in immune functioning such as heightened inflammatory responses (Slavich, 2020). This may explain why having a friendship with a close other is beneficial, but if the individual remains more broadly disconnected or out of step with their peer group (i.e., not socially integrated overall), they remain at risk for poor health outcomes as has been seen in other peer contexts (Mrug et al., 2012).

Remarkably few studies have examined the relationship between specific online social experiences and physical health; even fewer have focused specifically on markers of social integration or adherence to social norms. One issue is that social media use may involve many distinct activities, some of which tap into social integration (e.g., “liking” a friend’s picture or receiving a comment from a friend), and some of which do not tap into social integration (e.g., “liking” or clicking on links to articles that have been placed into one’s Facebook “news feed” by an algorithm). Studies that fail to distinguish between such activities may ultimately be measuring something more akin to “time spent on the internet” than social integration. These studies typically find social media use to be related to worse health outcomes (Lee et al., 2022). However, spending more time looking at screens and spending more time online have both been independently linked to poorer health outcomes (Do et al., 2013; Domingues-Montanari, 2017), so these studies may not provide much insight into the true effects of online social integration.

A handful of studies have examined number of Facebook friends in relation to physical health; this measure has some potential to serve as a proxy for social integration. These studies are mixed in their findings: two have found number of Facebook friends to be unrelated to physical health (Lima et al., 2017; Shakya & Christakis, 2017), while two have found number of Facebook friends to be positively associated with measures of physical health (Hobbs et al., 2016; Nabi et al., 2013). The mixed findings may be attributable to the limited utility of number of Facebook friends as a stand-alone measure of social support or social integration. For example, some individuals have a large objective number of Facebook “friends” but do not have strong social ties with nor fit in with these people, and in some cases, may not even know them.

Some research has been able to examine more fine-grained data about social media friendships (Hobbs et al. (2016)). This research was identified how many Facebook friendships a given participant had initiated (i.e., how many people the participant “friended”) and how many Facebook friendships other parties had initiated with the participant (i.e., how many people “friended” the participant). Participants who were friended more by others had lower mortality rates 2 years later. The authors hypothesized that receiving many friend requests was reflective of higher of social integration. The strong design of this study, including its longitudinal design and use of a coded, objective measure of online social experiences, underscore the study’s credibility. It seems that measures of online social integration may indeed hold important implications for physical health outcomes, but that no additional studies have sought to explore this possibility.

Similarly, there appears to be a lack of research that has sought to look at fitting in with social norms in a social media context in relation to physical health outcomes. This is somewhat surprising, as a growing literature explores norms of social media use. Researchers have been particularly interested in understanding the parameters of what is and is not appropriate to post on social media, and the social consequences of violating such norms of propriety. To this end, the term “faux pas” has been used by researchers to describe a certain type of inappropriate social media post: “items that might affect employer hiring decisions,” including photos and comments depicting or referencing nudity, firearms, illegal drugs, alcohol use, and sexual activities (Karl et al. (2010). In a study of adults of all ages, most participants reported being “very unlikely” to post such content (mean of 1.35 on a 1–5 likelihood scale; Roulin, 2014). In a study of college students, most participants reported being “very unlikely” or “unlikely” to post such content (mean of 1.51 on a 1–5 likelihood scale; Karl et al., 2010). Another study of college students found that participants were significantly more likely to engage in faux pas posting on Twitter than on Facebook and Instagram (Miller, 2020). However, most participants still reported being either “unlikely” or “neither likely nor unlikely” to post such content on Twitter (mean of 2.415 on a 1–5 likelihood scale). Thus, faux pas posting would indeed appear to be a non-normative activity among adults of all ages, particularly on Facebook.

Violating social norms via inappropriate online posting could hold negative social consequences for social integration. For example, one study that asked college students about the appropriateness of posting about romantic relationship drama on social media found that 74% of the participants considered such content inappropriate, and 28.9% of those participants would either block or unfriend the inappropriate posters (Roche et al., 2015). This translates to approximately 20% of the whole sample – meaning that potentially, 20% of all college students who come across such a post would remove the poster from their social network. Another study found that approximately 59% of college students would find posts about getting drunk to be inappropriate, and 88% find posting about vomiting due to drinking to be inappropriate (Wolfer, 2017). Moreover, 22.1% percent of participants reported that they would unfriend someone who posted about vomiting due to drinking. Crucially, these studies did not ask about participants’ judgments of people who have relationship drama or who get drunk in their personal lives – they focused on judgments of people who *post* about these things on social media. Collectively, this literature suggests that young adults who do not fit in with the broader peer group’s social norms about appropriate posting are at risk for low social integration. It therefore seems possible that the implications of faux pas posting would extend to the domain of physical health.

## Current Study

Although offline social experiences have been linked to physical health, it is unknown whether or not social experiences taking place on social media may also predict future physical health outcomes. To address these questions, the current study used multiple methods and a longitudinal design to examine the associations between online indicators of social integration – specifically, those found in posts on participants’ social media pages – assessed when participants were 21 years old, and multiple indicators of physical health, assessed when participants were 28 years old. It was hypothesized that general online social activity would not be predictive of future young adult physical health outcomes after controlling for baseline social, psychological, and physical variables (Hypothesis 1). As noted previously, it was thought that general activity on social media, such as number of friends or volume of activity, would be unlikely to provide insight into differential levels of social integration. It was next hypothesized that online social ties with peers, as evidenced on social media, would predict better future young adult physical health outcomes after controlling for baseline social, psychological, and physical variables (Hypothesis 2). As successful integration into the broader peer group seems to be key in promoting health, it was suspected that signifiers of online social ties with peers would promote positive health outcomes. Finally, it was hypothesized that posts that deviate from social norms – i.e., those containing “faux pas” content – would predict worse future young adult physical health outcomes after controlling for baseline social, psychological, and physical variables (Hypothesis 3). It was expected that the deviating from social norms for online posting would signify a lack of integration with the broader peer group, thereby leading to an increased risk of negative physical health outcomes.

## Methods

### Participants

Data are taken from a larger longitudinal investigation of adolescent social development in family and peer contexts. Participants included 138 young adults (59 men, 79 women) from whom interleukin-6 health data (described below) were collected, out of a total sample of 184 individuals. Participants were initially recruited from the seventh and eighth grades of a public middle school serving suburban and urban populations in the southeastern United States. Racially and socioeconomically, the sample was representative of the community from which it was drawn: 57% of participants identified themselves as White, 30% as Black/African American, and 13% as from other or mixed racial/ethnic groups. Participants’ parents reported a median family income in the $40,000–$59,999 range at the initial assessment in 1998–1999. At baseline, participants’ mean age was 13.35 (SD = 0.64), but data for this study were taken from two subsequent time points: at approximately age 21 (*M* age = 21.62, SD = 1.25), and age 28 (*M* age = 28.59 SD = 1.02). At age 21, participants completed questionnaires to assess their social competence and level of internalizing symptoms, and their physical attractiveness was observationally coded. Participants also consented to have their social media page (MySpace or Facebook) observationally coded for indicators of various social behaviors. At age 28, participants completed questionnaires about their health, consented to a blood draw to obtain levels of IL-6, and were measured and weighed to calculate their BMI.

### Attrition Analyses

Attrition analyses examined missing data for each variable available at baseline. Analyses found that women were more likely than men to participate at age 28 (*p* = 0.02), and that participants who had health data available at age 28 were also more likely to have deviant behavior in their online posted photos (*t* = −2.47, *p* = 0.01). Other than these differences, there were no other attrition effects. To best address potential biases due to missing data within waves, Full Information Maximum Likelihood (FIML) methods were utilized for all analyses, including all variables that were linked to future missing data (i.e., where data were not completely missing at random). These procedures have been found to provide the least biased estimates when all available data are used for longitudinal analyses (Arbuckle, 1996). No data is estimated or imputed in this procedure; rather, it simply accounts and corrects for biases due to missing data. As a result, all analyses reflect the entire sample for which there was data at age 28 (*n* = 138). Alternative longitudinal analyses using only those without any missing data yielded results that were substantially identical to those reported below.

### Measures

#### Social, Psychological, and Physical Control Measures (Age 21)

##### Self-rated social competence

Participants reported on their social competence at age 21 using the Harter Self-Perception Profile for Adolescents (Harter, 1988; McElhaney & Allen, 2001). Participants rated to what extent one of two contrasting stem items was more fitting for them, and then the degree to which that stem described them, on a 4-point scale from “not true at all” to “very true.” Examples of items from this scale included “Some people wish they had a really close friend to share things with, but other people do have a close friend to share things with”, “Some people are well liked by other people, but other people are not well liked by other people.” For these items, participants would thus first determine which part of a statement better applied to them (i.e. “Some people are well liked by others”, and then the degree to which that statement applied (i.e. “true” or “very true”). Responses were averaged to create a single rating for each scale. For the purposes of this study, the 5-item *competence in close friendships*, 4-item *social acceptance*, and 4-item *romantic appeal* subscales of the instrument were combined to create an overall measure of social competence. The overall social competence scale showed good internal consistency, yielding a Cronbach’s of 0.87.

##### Self-reported internalizing symptoms

Participants completed the Adult Self Report (Achenbach & Rescorla, 2003), a 126-item measure composed of internalizing, externalizing, substance use, attention problems, and thought problems subscales. The internalizing subscale, which is composed of 32 items assessing anxiety, depression, withdrawal, and somatic complaints, was used as a control measure in this study’s analyses. Examples of items from this scale include “I feel lonely,” I feel confused or in a fog,” and “I feel that no one loves me.” Items were scored on a three-point Likert scale where 0 = not true, 1 = somewhat or sometimes true, and 2 = very true or often true. Scores were summed with higher scores indicate greater levels of internalizing symptoms. This internalizing subscale showed good internal consistency, yielding a Cronbach’s α = 0.91.

##### Physical attractiveness

Participants’ physical attractiveness was coded from videotaped observations of participants and their closest same-gendered friend engaged in social interaction at age 21. During coding, half of the TV screen was covered so as to only show the participant. The audio was muted. Dynamic physical attractiveness (i.e. capturing both static appearance as well as movement and expressive behavior) was reliably coded using a naïve rater strategy on a scale of 1 to 7 with higher scores indicating greater physical attractiveness (Kopera et al., 1971; Patzer, 1985, Riggio et al., 1991). In a naïve coding system, coders are told to apply a lay understanding of the meaning of a given construct. The coding team (*n* = 8) was naïve to the purpose of this study, ethnically diverse, included both men and women, and achieved a reliability of ICC = 0.90 for their ratings.

### Coded Social Media Website Measures (Age 21)

At age 21 participants granted study staff access to their Facebook or MySpace profiles so that data could be collected from these social media platforms. If participants indicated they had a profile on both Facebook and MySpace, data were collected from the platform participants reported using most frequently (64% Facebook). An observational coding system was used to assess the quality of participants’ online social experiences on these platforms (Mikami et al., 2010, 2015). For all coded measures that involved posts received from peers, coders examined the 20 most recent messages from friends displayed on the participant’s web page (discrete posts individually stamped with the friend’s name, as the platform did not allow post strings or groups at the time of the coding). The 20 most recent posts were examined regardless of the number of different individuals who made them, and regardless of the time period over which the comments occurred. Thirty participants’ web pages, selected at random, were double-coded to provide an estimate of inter-rater reliability using intraclass correlation coefficients (ICCs; Shrout & Fleiss, 1979). Discrepancies between coders were handled by taking the average of coders’ scores. Social media data for the present study were collected and coded between February, 2008 and February, 2010.

### General Online Social Activity

#### Friend network size

The total number of individuals in a participant’s friendship network was displayed on and recorded from their Facebook or MySpace page (*M* = 306.45, *SD* = 255.67; ICC = 0.99).

#### Number of posts on page

At the time of coding, the total number of posts on a participant’s page since joining Facebook or MySpace was displayed on their page, and this was recorded (*M* = 229.67, *SD* = 303.80; Min. = 3, Max. = 2074; ICC = 0.99).

#### Number of different friends posting on page

The total number of different online friends posting messages on a participant’s page (within the 20 most recent posts) was recorded (*M* = 12.20, *SD* = 4.11; ICC = 0.96)

### Online Social Ties

#### Number of connection posts

Coders recorded the number of messages posted to the participant’s page indicating that the poster had seen or talked to the participant recently, planned to do so in the future, or which referenced or contained intimate information. Posts such as “I can’t wait to see you,” “It was great seeing you last night,” or “I’ll keep you posted about what’s going on at school,” would exemplify connection (*M* = 7.35, *SD* = 4.05; ICC = 0.81).

#### Number of different friends making connection posts

In addition to recording the total number of connection posts on the participant’s page, coders recorded the number of unique *individuals* who made connection posts (*M* = 5.28, *SD* = 2.62; ICC = 0.82).

#### Pictures with same-age peers

Coders examined pictures posted to participants’ pages for the presence of groups of same-age peers as a measure of social ties. Coders were instructed to consider individuals as same-age peers if they were likely to be within 2 years of age of the participant based on physical appearance. Photos posted by participants themselves, as well as by their friends, were considered. Coders considered participants’ photos as a total set and, using visual cues, assigned a global rating of 0–3. Participants’ pictures were rated “0” if there were no pictures posted on their page. Pictures were rated “1” if the clear majority of their pictures did not include any other same-age peers (such as landscapes). Pictures were rated “2” if the clear majority of pictures featured one or two same-age people. Pictures were rated “3” if the clear majority of pictures featured larger groups of same-age peers (*M* = 2.35, *SD* = 0.88; ICC = 0.75).

### Deviance from Social Norms about Online Posting

#### Number of posts with content deviating from social norms

Coders recorded the number of faux pas posts from friends on the participant’s page – those that would likely be considered embarrassing or judged as inappropriate if viewed by a parent, teacher, or employer. Examples of such posts were those which contained explicit profanity and indirect or direct references to alcohol use, drugs, delinquency, or sex (*M* = 1.67, *SD* = 1.31; ICC = 0.77).

#### Number of different friends posting content deviant from social norms

In addition to coding the total number of posts with content deviating from norms on each participant’s page, coders recorded the number of unique *individuals* who made such posts (*M* = 1.40, *SD* = 1.31; ICC = 0.78).

#### Pictures with behavior deviating from social norms

Coders examined pictures posted to participants’ pages to determine what proportion of pictures showed the target participant engaging in behavior that might be embarrassing or judged as inappropriate if viewed by a parent, teacher, or employer (i.e. authority figure). Photos posted by participants themselves, as well as by their friends, were considered. Coders considered participants’ photos as a total set and, using visual cues, assigned a global rating of 0–3. Participants’ pictures were rated “0” if there were no pictures posted on their page. Participants’ pictures were rated “1” if the clear majority of their pictures did *not* contain them engaging inappropriate behavior. For example, a few pictures of the target participant and/or friends hanging out and legally drinking beer would not be considered inappropriate. Pictures were rated “2” if some of the pictures posted showed the participant engaging in behaviors that *might* be considered inappropriate if viewed by a parent or authority figure (e.g., drinking games, dressing provocatively). Pictures were rated “3” if there was at least one picture that showed the participant and/or others engaging in clearly inappropriate behavior. Such behaviors included (but were not limited to) vandalism, passing out or vomiting from drinking, nudity, or other generally out-rageous or overtly sexual actions (*M* = 1.57, *SD* = 0.91; ICC = 0.76).

### Health Measures (Age 28)

#### Interleukin-6 (IL-6)

This cytokine associated with inflammation, and when found in the bloodstream at higher levels, a marker of potentially dysregulated immune functioning. Approximately 20 ml of blood were collected and treated with EDTA (to prevent clotting) to determine circulating concentrations of IL-6. Plasma was separated via centrifugation, aliquoted, and stored at −80 8 C. IL-6 was measured by ELISA (limit of detection ¼ 0.3 pg/ml; R&D Systems, San Diego, CA). Intraassay and interassay coefficients of variation (%CV) were 3.6 and 8.6% for IL-6. Resulting scores were then log-transformed, as is typical with this measure, to address skewness.

#### Subjective sleep quality

Subjective sleep quality was assessed using The Pittsburgh Sleep Quality Index (Buysse et al., 1988), a self-report questionnaire which assesses sleep quality and disturbances over a 1-month time interval. Subjective sleep quality is one of seven subscales of the measure and is derived from participants’ rating of a single-item asking about their sleep quality overall during the past month on a 0–3 scale, with higher scores indicating better sleep quality.

#### Physical functioning

Participant physical functioning was assessed using a Health Experience Questionnaire adapted from the RAND 36-item Health Survey version 1.0 (Ware, & Sherbourne, 1992), which was designed to assess eight health domains: physical functioning, bodily pain, role limitations due to physical health, role limitations due to emotional problems, emotional well-being, social functioning, fatigue, and general health problems. The physical functioning subscale includes participants’ report of whether and to what extent their health limits their ability to perform 10 different everyday activities such as vigorous activities (e.g. running) and bending, kneeling, or stooping. All items are scored on a 1–3 scale with high scores defining a more favorable health state. The physical functioning subscale showed good internal consistency, yielding a Cronbach’s alpha of 0.93.

#### BMI

Height (in meters) and weight (in kilograms) were assessed with light clothing, and BMI was calculated using the standard formula BMI ¼ weight/height^2.

## Results

### Preliminary Analyses

#### Univariate and correlational analyses

Means and standard deviations for all primary variables are presented in Table 1. For descriptive purposes, correlations were examined between all key variables of interest and are presented in Table 2. These analyses revealed that gender and family income had significant associations with several primary variables. Women were more likely to have a greater number of posts from friends on their social media webpage (*r* = 0.24, *p* < 0.05), as well as a greater number of posts featuring connection comments as compared to men (*r* = 0.23, *p* < 0.05). Women also had fewer posts containing deviant content (*r* = −0.28, *p* < 0.01) and fewer friends posting comments with deviant content (*r* = −0.24, *p* < 0.05), than men. Participants with higher household income levels had a greater number of posts featuring connection (*r* = 0.22, *p* < 0.05), and a greater number of different friends making connection posts (*r* = 0.33, *p* < 0.01). Higher income was also associated with lower levels of IL-6 (*r* = −0.27, *p* < 0.001).

Preliminary analyses also investigated possible associations between predictor, control, and outcome variables. Results of these correlational analyses revealed several positive significant associations between online social media variables at age 21 and physical health outcome variables at age 28 (see Table 2). Outcome variables were moderately correlated with one another, suggesting these variables represented related but not redundant constructs within the domains of physical health functioning in young adulthood. The control variables of social competence, internalizing symptoms, and physical attractiveness assessed at age 21 were uncorrelated with one another, with the exception of a correlation between social competence and internalizing symptoms (*r* = −0.59, *p* < 0.001). As hypothesized, there were several strong correlations between these control variables and physical health outcomes assessed at age 21. Social competence was associated with better sleep quality (*r* = 0.30, *p* < 0.001), whereas internalizing symptoms were associated with worse sleep quality (*r* = −0.25, *p* < 0.01). Higher ratings of participant physical attractiveness were associated with lower levels of IL-6 (*r* = −0.39, *p* < 0.001), better physical functioning (*r* = 0.24, *p* < 0.01), and lower BMI (*r* = −0.54, *p* < 0.001) at age 28.

### Primary Analyses

Hypotheses were tested using hierarchical regression modelling to assess the unique contributions of different aspects of online social integration to outcomes related to young adult physical health after controlling for relevant social, psychological, and physical participant characteristics. Analyses considered the extent to which general online social activity, social ties, and deviance from peer group norms about posting at age 21 would predict future levels of IL-6, sleep quality, physical functioning, and BMI at age 28. Because of the high level of multicollinearity, online social integration variables were entered separately (rather than as a block) into individual regression models. Thus, for all models, participant gender and household income were entered into the model together at Step 1, and social competence, internalizing symptoms, and physical attractiveness at age 21 were entered as a block at Step 2. At Step 3, the relevant online social integration variable was entered. Tables 3–4 show the effects of and variance explained by Steps 1 and 2 at entry to the model, as well as the final unique effect of and variance explained by each online social integration variable after controls were entered. Power estimates indicate that 80% power would be obtained for standardized estimates equal to or greater than 0.21

#### Hypothesis 1

*General online social activity will not be predictive of future young adult physical health outcomes after controlling for baseline social, psychological, and physical variables*. Initial correlational analyses found that having a greater number of online friends was associated with better subjective sleep quality (*r* = 0.26, *p* < 0.05). Moreover, receiving wall post comments from a greater number of friends was correlated with lower levels of IL-6 (*r* = −0.25, *p* < 0.05), better sleep quality (*r* = 0.26, *p* < 0.05), and better *p*hysical functioning (*r* = 0.29, *p* < 0.01). Although these trends were also evident in regression models, they all dropped below conventional levels of significance after including the control variables. No other significant effects emerged.

#### Hypothesis 2

*Online social ties with peers, as evidenced on social media, will predict better future young adult physical health outcomes after controlling for baseline social, psychological, and physical variables*. Correlations showed that having a greater number of connection posts was associated with lower IL-6 levels (*r* = −0.23, *p* < 0.05). Having a greater number of friends making connection posts was associated with lower IL-6 levels (*r* = −0.41, *p* < 0.001), better sleep quality (*r* = 0.26, *p* < 0.05), better physical functioning (*r* = 0.26, *p* < 0.05), and lower BMI (*r* = −0.33, *p* < 0.01). Having same-aged peers featured in more posted pictures was also associated with lower IL-6 levels (*r* = −0.23, *p* < 0.05) and lower BMI (*r* = −0.38, *p* < 0.001). After entering control variables in regression models, the number of connection posts was no longer a significant predictor of any outcomes. Having a greater number of friends making connection posts continued to predict lower IL-6 levels (β = −0.23 p < 0.05) and better sleep quality (β = 0.29, p < 0.01). Having same-aged peers featured in more posted pictures remained a significant *p*redictor of lower BMI (β = −0.25, p < 0.01). See Table 3.

#### Hypothesis 3

*Posts that deviate from social norms – i.e., those containing “faux pas” content – will predict worse future young adult physical health outcomes after controlling for baseline social, psychological, and physical variables*. There were significant correlations between having a greater number of friends making posts deviant from online social norms and higher IL-6 levels (*r* = −0.22, *p* < 0.05), worse sleep quality (*r* = −0.25, *p* < 0.05), and worse physical functioning (*r* = −0.24, *p* < 0.05). Having posted pictures that were more deviant from social norms was associated with lower IL-6 levels (*r* = −0.23, *p* < 0.05) and lower BMI (*r* = −0.22, *p* < 0.05). After entering control va*r*iables in regression models, both a greater number of deviant comments (β = 0.24 *p* < 0.05) and a greater number of posts that deviate from social norms (β = 0.23 *p* < 0.05) predicted higher levels of IL-6. A greater number of posts that deviate from social norms also predicted worse sleep quality (β = −0.29 *p* < 0.01) and worse physical functioning (β = −0.34 *p* < 0.01). Having pictures that were deviant from social norms was no longer a significant predictor of any outcomes when entered into a regression model. See Table 4.

#### Post Hoc Analyses

Although the control variables of social competence, internalizing symptoms, and physical attractiveness explained between 9–33% of variance in study outcomes, post hoc analyses were also conducted with the inclusion of a retrospective physical health variable regarding participants’ history of serious illness prior to age 18. Participants reported on the presence versus absence of 43 distinct significant health problems that were first experienced prior to age 18 years and which had led, at some point, to hospitalization (*M* = 0.17; *SD* = 0.44). The measure thus provides a baseline marker of potential pre-existing health difficulties and has been found to previously predict current adult health quality (Allen, Uchino, & Hafen, 2015). This measure of prior illness was not correlated with social competence, internalizing problems, nor physical attractiveness at age 21. It was also not correlated with IL-6, physical functioning, nor BMI at age 28; the only significant positive correlation was with worse sleep quality (*r* = 0.20, *p* = 0.02) at age 28.

When included as an additional covariate in final regression models with significant online predictors, history of prior illness was not a significant predictor of any outcome at age 28. In these models, having a greater number of friends making connection posts continued to significantly predict better sleep quality (*β* = 0.25, *p* < 0.05), and lower BMI (*β* = −0.25, *p* < 0.01). The previous significant association with lower IL-6 (*β* = −0.23, *p* < 0.05) dropped just below statistical significance (*β* = −0.19, *p* = 0.09) when prior illness was included; however, this seemed to be a result of a slightly amplified effect of physical attractiveness (*β* = 0.33 vs. *β* = 0.30), as history of prior illness itself was not predictive of IL-6 (*β* = 0.01, *ns*).

Similarly, when prior illness was included, posts containing content that deviates from social norms for posting (*β* = 0.23, *p* < 0.05) and a greater number of friends making such posts on the participant’s page (*β* = 0.21, *p* < 0.05) continued to predict higher IL-6 levels. A greater number of posts with such content also continued to predict worse sleep (*β* = −0.27, *p* < 0.01) and worse physical functioning (*β* = −0.36, *p* < 0.01).

Finally, we examined the potential for externalizing behaviors and alcohol use to explain the study’s findings. When externalizing behaviors and alcohol use (a measure of number of alcoholic drinks consumed per week) were added into models, respectively, having a greater number of friends making connection posts remained a predictor of lower IL-6 levels (*β* = −0.25, *p* < 0.05; *β* = −0.26, *p* < 0.05) and fewer sleep problems (*β* = −0.27, *p* < 0.05; *β* = −0.28, *p* < 0.05). Having a greater number of friends making posts with content deviating from peer norms predicted higher IL-6 levels (*β* = 0.24, *p* = 0.01; *β* = 0.24, *p* = 0.01), greater sleep problems (*β* = −0.25, *p* < 0.05; *β* = −0.28, *p* < 0.01), and worse physical functioning (*β* = −0.33, *p* < 0.01; *β* = −0.35, *p* < 0.01). Having more pictures with same-age peers also continued to predict lower BMI in both conditions (*β* = −0.25, *p* < 0.05; *β* = −0.25, *p* < 0.05).

## Discussion

Although offline social experiences have been linked to markers of physical health, social experiences taking place online during adolescence have yet to be considered as predictors future physical health outcomes, particularly after controlling for other likely explanatory factors. This study examined social media posts received from peers as predictors of participants’ future physical health outcomes (e.g., Interleukin-6 (inflammation), sleep problems, problems with physical functioning, and BMI) at age 28. The results of this study found that several indicators of social integration observed in posts on participants’ social media pages in their early 20 s were strong predictors of their long-term physical health across a range of outcomes 7 years later. Importantly, these results remained after accounting for potential social, psychological, and physical confounds. The results indicated two key features of the types of posts that were most predictive of future physical health. Predictive posts contained clear markers of either social ties with peers or potential deviance from peer norms about posting. Second, it was the number of different individuals making such posts on the participant’s, rather than the sheer frequency of these posts, that were most often predictive. In contrast, more general markers social media activity such as friend network size, overall number of posts received, or even the number of different individuals posting (without regard to what they were posting) were *not* ultimately predictive of future physical health outcomes after accounting for controls. Each of these findings is discussed in turn below.

Generally speaking, social media posts from friends that displayed evidence of a *social tie or personal connection* between the participants and friends, either through their verbal content or via pictures, were correlated with lower levels of IL-6, better sleep quality, and lower BMI. The current study also found that faux pas posts - featuring content *deviating from peer norms about posting* - were associated with participants’ future higher levels of IL-6, worse sleep quality, and lower physical functioning. This mirrors findings from a large body of research underscoring the importance of social integration for physical health. while extending these indicators of social integration to the online domain.

Indeed, social media use has become ubiquitous for young people and social integration in this domain has likely become a social competency unto itself (Politte-Corn et al., 2023). Youth who are successfully integrated in the online domain (as shown by having strong social ties on social media) are likely to reap benefits for their physical health, because so much of their social interaction occurs online. Conversely, a failure to integrate socially in this context (as shown by having content that deviates from peer norms about social media posts) may put individuals at risk of alienation from their peers, with potentially significant consequences for their health and well-being. Young adults may also use social media as a context in which to enact the same relationship patterns (healthy or unhealthy) that they display in offline settings (Mikami & Szwedo, 2011). Research has suggested that offline and online social skills are related, though not identical (*r* = 0.18; Resnik & Bellmore, 2019); this research also found positive correlations between online social skills and a sociometric measure of social media acceptance, and additional positive correlations between social media acceptance and broader peer acceptance.

However, when entered into regression models including possible alternative explanatory variables, a particular feature of the most predictive types of social media posts emerged. Although it might have been expected that simply receiving more posts indicating social ties, or conversely, more posts that deviated from peer norms, would strengthen predictions to outcomes, nearly all correlations between such “frequency” variables and outcomes were diminished in regression analyses. Instead, the most predictive variables were often those which captured the *number of different individuals* making a particular type of post.

When considering posts that indicate social ties (connection posts), these results are perhaps suggestive of the greater importance of broader social integration/acceptance in the online domain relative to ties with one or two particular individuals for predicting adaptative outcomes. This is an interesting possibility given recent evidence that a stronger friendship with just one individual seems generally to serve as a more robust predictor of long-term adjustment relative to self-perceived broader social acceptance (Narr et al., 2019). Because online social media essentially maintains a historical record of recent and previous social experiences with peers that is easily reviewable, it may be that in this domain social ties are more likely to be considered broadly than individually. Perhaps benefits are gained when individuals are able to look through a social media feed and recognize examples of social ties with many others among their posts rather than a few. Indeed, research has found that while a measure of close friendship quality with another person contributed indirectly to later physical health quality, it was teens’ ability to understand, adapt to, and fit in with the norms of their larger peer social group that directly predicted future health quality (Allen et al., 2015).

Notably, however, receiving posts that deviated from peer norms from a greater number of individuals did *not* similarly predict enhanced physical outcomes, but rather predicted the opposite: poorer physical health. Interacting with a greater number of individuals online thus seems to cut two ways: positively if done in a way that demonstrates or strengths social ties, or negatively if done in a way that promotes social deviancy relative to expected peer norms for posting. Although it is unclear if interacting with more individuals in a socially deviant way online has any psychological benefits for teens (some research has suggested youth with a deviant friend have lower friendship quality overall (Poulin, Dishion, & Haas, 1999), though some has shown that while such youth have similar levels of depression as friendless youth, they are less lonely (Brendgen, Vitaro, & Bukowoski, 2000), the data point to long-term problems in the physical domain, at least for young adults. Even if there may be some short term social or psychological benefits to such interactions, the nature of these interactions is unlikely to be accepted more broadly by the peer group (Roche et al., 2015), which could create difficulties in forming and maintaining appropriate relationships with others in the future, such as with romantic partners or co-workers. This may be particularly problematic when considering physical health outcomes, as the benefits of sustained romantic relationships for physical health are well-established, and interpersonal difficulties in the workplace might make it more difficult to sustain employment necessary to help pay for physical health care if needed (Johnson, Backlund, Sorlie, & Loveless, 2000).

Although these data help identify the types of social media posts that may be predictive of future physical health outcomes, the potential mechanisms of these predictions are somewhat less clear. The findings related to posts showing social ties (connection posts) are consistent with the idea of how building a system of positive social support may confer health benefits. Ample research has identified both loneliness (Holt-Lundstad et al., 2010) and stress (Glaser & Kiecolt-Glaser, 2005) as predictive of physical health problems. Indicators of social ties online were often suggestive of real-world interactions between friends, suggesting that such posts may be indicators that individuals are spending time with others, thereby potentially reducing feelings of loneliness. As suggested above, the fact that social ties are indicated and available online also means that individuals may be able to access these ties even when they are alone (indeed, that may be when they are most likely to). Thus, individuals may not necessarily have to feel likely they are alone very often, as they can both be in touch with friends at almost any time via social media and can also review their messages from friends and be reminded of the ties they have with them (Allen et al., 2014).

Taken further, to the extent that connection posts do reflect more intimate friendships, individuals who receive such posts may truly be able to call upon those friends as sources of support when stressed. These possibilities suggest that there may be psychological or social features that help account for the associations between connection posts and physical health outcomes. Interestingly, individuals receiving connection posts from a greater number of friends neither reported being less troubled by internalizing symptoms, nor having greater social competence, as compared to participants receiving these posts from fewer friends online, and their predictive effects held after considering these variables in regression models. This suggests there may be alternative processes to consider with regard to how receiving such posts might be predictive of future health. Indeed, they may be more specific, in terms of actually being able to call on others for emotional or practical support, or feeling positive about the state of one’s friendships in a way that promotes physical health through stress reduction. On this point, it should be noted that having more pictures posted to their social media page that featured same-age peers was predictive of a lower future BMI, even after controlling for baseline levels of physical attractiveness that were highly negatively correlated with BMI. This suggests the possibility that friendships indicating social ties may also feature more in-person interactions, which has the potential to include behaviors that could promote positive physical health via in-person activity.

As suggested above, although having friends who post content that deviates from peer norms may have some immediate psychosocial benefits (e.g. less loneliness), there may be limitations to it that contribute to negative physical health outcomes. Notable in the data was that receiving a greater number of posts that deviated from peer norms from others was negatively correlated with online friendship network size and showed a trend toward negative correlations with number of connection posts received from others and pictures featuring same-age peers. This suggests that although such individuals may have others with whom to communicate, they may have a smaller social network to draw from overall and be less likely to share more social ties with their friends online. In the long term, this type of online social experience may play out oppositely from that experienced by individuals with many social ties; their friends may be less reliable sources of support, and they may lack the skills needed to even request such support in the first place. Individuals who engage in more deviant conversations online may also be more likely to engage in riskier behaviors that could contribute to negative health consequences (Dishion et al., 1996). For example, previous research has identified deviant behavior in photos online as directly predictive of individuals’ problematic alcohol use (Szwedo et al., 2012).

Importantly, more general markers of social media activity such as friend network size, overall number of posts received, or number of different individuals posting overall, were ultimately *not* predictive of future physical health outcomes in regression models. Initial correlations suggested some promise for these variables, with greater friendship network size correlated with better sleep quality, and the number of different individuals posting overall correlated with every outcome in a way as to indicate more positive physical health. However, it is suspected that these associations may have been driven by receiving a larger number of connection posts in particular from among the larger number overall. Such results indicate the importance of looking beyond general indicators of online activity and instead toward specific types of interactions that youth are having online.

Beyond limitations noted above, several others merit consideration. First, this study was correlational in nature, precluding causal conclusions from being drawn from any of its findings. It is possible that other processes unaccounted for in the present study might help improve understanding of the observed associations between online social media posts and future health outcomes. Moreover, there are likely other relevant qualities of posts that could be predictive of future health outcomes (e.g., emotional support received, aggressive behavior). Although the coding system captured observed posts on the participants’ social media page (from peers and from the participant), at the time online data collection occurred there was not an easily accessible way to see what participants were posting on friends’ pages. It would be helpful to have a sense of whether or not there is evidence of a social tie on both ends (reciprocation), and if this is a significant predictor of health outcomes. Similarly, it would be useful to see if participants themselves are making posts on friends’ pages that deviated from peer norms for posting. Individuals’ own behaviors online may be the strongest predictors of their future health. More recent iterations of social media also allow for private and backchannel messaging, which undoubtedly contributes to individuals’ overall well-being, but is not typically available for others to see.

Another limitation was that we did not have baseline physical health information to include in analyses. As a result, we selected a range of social, psychological, and physical variables that have been linked to physical health in previous studies as controls. We also further probed analyses by including a post-hoc variable of historical prior illness, which did not significantly alter the pattern of results (nor did externalizing behavior or alcohol use). Still, baseline health assessments would help strengthen the study’s ability to suggest that predictors were indicative of change over time in physical health. It is also possible that participants’ actual offline behaviors (e.g., poor sleep hygiene for sleep problems) might mediate associations between online predictors and health outcomes. Future studies would benefit from greater consideration and inclusion of variables representing such behaviors to better understand the contribution of online behaviors relative to offline behaviors. The relative youth of the sample is also relevant, given that many participants may not begin to develop more significant health problems until later in their lives. As such, we included broad markers of physical health that might be more relevant to a broad age-range, including stress (IL-6), sleep, overall physical functioning, and BMI. Future studies would benefit by exploring additional health outcomes that might include more specific health behaviors (e.g., cigarette smoking, alcohol use), or other health factors (e.g. blood pressure, cholesterol), and possibly at older ages. Finally, though this study aimed to use more objective (i.e. coded) measures when possible, many control and outcome variables were self-reported and potentially subject to biases (i.e. social desirability).

## Conclusion

One of the most important implications from this study is that how socially integrated young adults are with their peers online deserves careful attention, not only for its potential social and psychological consequences, but also for its potential long-term physical health consequences. The results of this study corroborate, and extend into the online domain, recent research that has identified the quality of adolescent peer relationships as key predictors of physical health. Indeed, peer relationships on social media may be an even more powerful mechanism for promoting the types of relationships that will help individuals thrive or struggle as they enter early adulthood as compared to in-person communication. For example, the ever-available nature of online communication via social media provides ongoing opportunities to build social ties characterized by intimacy and support. Even when individuals are not actively communicating with others, they are able to review previous interactions and derive positive emotional experiences from doing so. However, the online domain also allows individuals the opportunity to more easily build relationships with others who may not ultimately help them become more appropriately functional adults. Much of the time, peer relationship dynamics operate outside the view of parents and other important adults, so it may be difficult for outsiders to fully appreciate the nature of these relationships and to know if they are healthy or problematic. Given their importance to long-term development, parents and others might be well-served to look at the at the social integration that youth are experience online, and to encourage healthy communication and social ties while expressing concern for more inappropriate posting, as it is becoming increasing clear that even virtual interactions have very real long-term implications.

## Figures and Tables

**Table 1:** Descriptive statistics for all study variables

Variable	*N*	Mean	*SD*	Min	Max	*r* with Gender	*r* with Income
Gender (59 male, 79 female)	138	–	–	1=male	2=female	–	–
Income	135	40–60 K/year	–	<$5 K/yr	>$60 K/yr	−0.15	–
Social Competence (21)	120	42.84	7.24	22	52	0.00	−0.01
Internalizing Symptoms (21)	123	11.14	11.10	0	56	0.09	0.10
Physical Attractiveness (21)	109	3.51	0.95	1.4	6.1	0.03	0.15
# of Facebook Friends (21)	84	306.45	255.67	11	1143	0.02	0.21
# of Posts From Friends (21)	74	229.67	303.80	3	2074	0.24*	0.14
# of Posts From Diff. Friends (21)	85	12.20	4.11	1	20	−0.03	0.19
# of Connection Posts (21)	85	7.35	4.05	0	17	0.23*	0.22*
# of Connection Posts from Diff. Friends (21)	85	5.28	2.62	0	11	0.14	0.33**
Same-Age Peers in Photos (21)	85	2.35	0.88	1	3	0.03	0.18
# of Deviant Posts (21)	77	1.67	0.90	0	3	−0.28**	−0.04
# of Deviant Posts from Diff. Friends (21)	77	1.40	1.31	0	4	−0.24*	−0.01
Pictures with Deviant Content (21)	85	1.57	0.91	1	3	−0.01	0.12
Interleukin-6 (28)	138	0.21	0.87	−1.56	2.72	0.14	−0.27***
Sleep Quality (28)	130	0.97	0.62	0	3	0.06	0.00
Physical Functioning (28)	128	91.68	16.53	25	100	−0.11	0.11
BMI (28)	126	28.24	8.87	16.43	68.88	0.07	−0.06

* *p* < 0.05,

** *p* < 0.01,

*** *p* < 0.001

**Table 2:** Correlations between primary study variables

	1	2	3	4	5	6	7	8	9	10	11	12	13	14	15
1. Social Competence (21)	–														
2. Internalizing Symptoms (21)	−0.59***	–													
3. Physical Attractiveness (21)	0.01	0.10	–												
4. # of Facebook Friends (21)	0.15	−0.15	0.09	–											
5. # of Posts From Friends (21)	0.05	−0.03	0.22	0.75***	–										
6. # of Posts From Diff. Friends (21)	0.18	−0.21	0.25*	0.55***	0.44***	–									
7. # of Connection Posts (21)	−0.13	−0.03	0.30**	0.08	0.12	0.13	–								
8. # of Connection Posts from Diff. Friends (21)	−0.08	−0.09	0.39***	0.30***	0.31**	0.42***	0.83***	–							
9. Same-Age Peers in Photos (21)	−0.14	0.10	0.20	0.14	0.16	0.13	0.16	0.29**	–						
10. # of Deviant Posts (21)	−0.02	−0.11	−0.01	−0.18	−0.03	0.02	−0.16	−0.20	−0.19	–					
11. # of Deviant Posts from Diff. Friends (21)	−0.01	−0.05	0.00	−0.31***	−0.13	−0.03	−0.15	−0.20	−0.18	0.72***	–				
12. Pictures with Deviant Content (21)	−0.10	0.19	0.20	0.06	0.16	0.04	−0.07	0.10	0.57***	0.03	0.02	–			
13. Interleukin–6 (28)	0.12	−0.07	−0.39***	−0.13	−0.06	−0.25*	−0.23*	−0.41***	−0.23*	0.21	0.22*	−0.22*	–		
14. Sleep Quality (28)	0.30***	−0.25**	0.18	0.26*	0.19	0.26*	0.08	0.26*	0.07	−0.12	−0.25*	−0.03	−0.20*	–	
15. Physical Functioning (28)	−0.03	−0.10	0.24**	0.10	0.04	0.29**	0.09	0.25*	0.03	−0.08	−0.24*	−0.05	−0.28***	−0.17*	–
16. BMI (28)	0.11	0.00	−0.54***	−0.03	−0.07	−0.21	−0.16	−0.34***	−0.38***	0.06	0.05	−0.23*	0.58***	0.14	−0.35***

* *p* < 0.05,

** *p* < 0.01,

*** *p* < 0.001

**Table 3:** Regression analyses examining social ties in posts as predictors of physical health outcomes

	Interleukin-6 (28)			Sleep Quality (28)			Physical Functioning (28)			BMI (28)		
	β	*ΔR* ^*2*^	*R* ^2^	β	*ΔR* ^*2*^	*R* ^2^	β	*ΔR* ^*2*^	*R* ^2^	β	*ΔR* ^*2*^	*R* ^2^
**Step I**		0.09**	0.09**		0.01	0.01		0.02	0.02		0.01	0.01
Gender	0.10			−0.07			−0.10			0.06		
Income	−0.26***			−0.01			0.09			−0.06		
**Step II**		0.16*	0.25***		0.12**	0.13*		0.09**	0.11		0.33***	0.34***
Social Competence (21)	0.17			0.20			−0.17			0.16		
Internalizing Symptoms (21)	0.08			−0.14			−0.23*			0.13		
Physical Attractiveness (21)	−0.39***			0.19*			0.26**			−0.58***		
**Model 1 Step III**		0.00	0.25***		0.00	0.13*		0.00	0.11		0.00	0.34***
Number of Connection Posts (21)	−0.06			0.08			0.01			0.08		
**Model 2 Step III**		0.03*	0.28***		0.05**	0.18**		0.02	0.13*		0.00	0.34***
Number of Different Friends	−0.23*			0.29**			0.17			−0.05		
Making Connection Posts (21)												
**Model 3 Step III**		0.01	0.26***		0.01	0.14*		0.00	0.11		0.05***	0.39***
Pictures with Same-Age Peers (21)	−0.13			0.11			−0.02			−0.25**		

* *p* < 0.05,

** *p* < 0.01,

*** *p* < 0.001

**Table 4:** Regression analyses examining faux pas posts as predictors of physical health outcome

	Interleukin-6 (28)			Sleep Quality (28)			Physical Functioning (28)			BMI (28)		
	β	*ΔR* ^*2*^	*R* ^2^	β	*ΔR* ^*2*^	*R* ^2^	β	*ΔR* ^*2*^	*R* ^2^	β	*ΔR* ^*2*^	*R* ^2^
**Step I**		0.09**	0.09**		0.01	0.01		0.02	0.02		0.01	0.01
Gender	0.10			−0.07			−0.10			0.06		
Income	−0.26***			−0.01			0.09			−0.06		
**Step II**		0.16*	0.25***		0.12**	0.13*		0.09**	0.11		0.33***	0.34***
Social Competence (21)	0.17			0.20			−0.17			0.16		
Internalizing Symptoms (21)	0.08			−0.14			−0.23*			0.13		
Physical Attractiveness (21)	−0.39***			0.19*			0.26**			−0.58***		
**Model 1 Step III**		0.04***	0.29***		0.02	0.15*		0.03*	0.14*		0.00	0.34***
Number of Deviant Posts (21)	0.24*			−0.16			−0.19			0.06		
**Model 2 Step III**		0.04***	0.29***		0.07***	0.20**		0.10***	0.21**		0.00	0.34***
Number of Different Friends Making Deviant Posts (21)	0.23*			−0.29**			−0.34**			0.03		
**Model 3 Step III**		0.01	0.26***		0.00	0.13*		0.00	0.11		0.00	0.34***
Pictures with Deviant Content (21)	−0.16			0.01			−0.11			−0.10		

* *p* < 0.05,

** *p* < 0.01,

*** *p* < 0.001

## Data Availability

This manuscript’s data will not be deposited.

## References

[R1] Achenbach TM, & Rescorla LA, (2003). Manual for the ASEBA adult forms & profiles.

[R2] Allen JP, Uchino BN, & Hafen CA (2015). Running With the Pack: Teen Peer- Relationship Qualities as Predictors of Adult Physical Health. Psychological Science, 26(10), 1574–1583. 26290522 10.1177/0956797615594118 PMC4698963

[R3] Allen JP, Loeb EL, Tan J, Davis AA, & Uchino B (2021). Adolescent relational roots of adult blood pressure: A 14-year prospective study. Development and Psychopathology, 1, 11. 10.1017/s0954579421000419 PMC9836075 36643089

[R4] Allen KA, Ryan T, Gray DL, McInerney DM, & Waters L (2014). Social media use and social connectedness in adolescents: The positives and the potential pitfalls. The Educational and Developmental Psychologist, 31(1), 18–31.

[R5] Arbuckle JL (1996). Full information estimation in the presence of incomplete data. In Schumaker GAMRE (Ed.), Advanced structural modeling: Issues and techniques (pp. 243–277). Erlbaum.

[R6] Auxier B, & Anderson M (2021). Social media use in 2021: a majority of Americans say they use YouTube and Facebook, while use of Instagram, Snapchat and TikTok is especially common among adults under 30. Pew Research Center.

[R7] Beckes L, & Coan JA (2011). Social baseline theory: The role of social proximity in emotion and economy of action. Social and Personality Psychology Compass, 5(12), 976–988.

[R8] Boye B, Jahnsen J, Mokleby K, Leganger S, Jantschek G, Jantschek I, & Lundin KEA (2008). The INSPIRE study: are different personality traits related to disease-specific quality of life (IBDQ) in distressed patients with ulcerative colitis and Crohn’s disease? Inflammatory Bowel Diseases, 14(5), 680–686. 18509900 10.1002/ibd.20367

[R9] Brendgen M, Vitaro F, & Bukowski MW (2000). Deviant friends and early adolescents’ emotional and behavioral adjustment. Journal of Research on Adolescence, 10(2), 173–189.

[R10] Buysse DJ, Reynolds CF III, Monk TH, Berman SR, & Kupfer DJ (1988). The Pittsburgh sleep quality index: A new instrument for psychiatric practice and research. Psychiatry Research, 28(2), 193–213. 10.1016/0165-1781(89)90047-4 2748771

[R11] Czajkowski SM, Arteaga SS, & Burg MM (2022). Social support and cardiovascular disease. In Handbook of Cardiovascular Behavioral Medicine (pp. 605–630). New York, NY: Springer New York.

[R12] Dishion TJ, Spracklen KM, Andrews DW, & Patterson GR (1996). Deviancy training in male adolescent friendships. Behavior therapy, 27(3), 373–390.

[R13] Do YK, Shin E, Bautista MA, & Foo K (2013). The associations between self-reported sleep duration and adolescent health outcomes: what is the role of time spent on Internet use? Sleep Medicine, 14(2), 195–200. 23068781 10.1016/j.sleep.2012.09.004

[R14] Domingues‐Montanari S (2017). Clinical and psychological effects of excessive screen time on children. Journal of Paediatrics and Child Health, 53(4), 333–338. 28168778 10.1111/jpc.13462

[R15] Ferguson G, Sliwinski M, & Scott S (2020). Relationship Between Health and Well-Being and Adherence to Social Norms Across Adulthood. Innovation in Aging, 4(Supplement_1), 312–312.

[R16] Glaser R, & Kiecolt-Glaser J (2005). Stress-induced immune dys-function: implications for health. Nature Reviews Immunology, 5, 243–251. 10.1038/nri1571. 15738954

[R17] de Grey RG, Uchino BN, Trettevik R, Cronan S, & Hogan JN (2018). Social support and sleep: A meta-analysis. Health Psychology, 37(8), 787. 29809022 10.1037/hea0000628

[R18] Gross EB, & Medina-DeVilliers SE (2020). Cognitive processes unfold in a social context: a review and extension of social baseline theory. Frontiers in Psychology, 11, 378. 32210891 10.3389/fpsyg.2020.00378 PMC7076273

[R19] Harter S (1988). Manual for the self-perception profile for adolescents. Denver, CO: University of Denver.

[R20] Ho CY (2016). Better health with more friends: the role of social capital in producing health. Health Economics, 25(1), 91–100. 25431183 10.1002/hec.3131

[R21] Hobbs WR, Burke M, Christakis NA, & Fowler JH (2016). Online social integration is associated with reduced mortality risk. Proceedings of the National Academy of Sciences, 113(46), 12980–12984. 10.1073/pnas.1605554113 PMC5135317 27799553

[R22] Holt-Lunstad J, Smith TB, & Layton JB (2010). Social relationships and mortality risk: a meta-analytic review. PLoS medicine, 7(7), e1000316. 20668659 10.1371/journal.pmed.1000316 PMC2910600

[R23] Johnson NJ, Backlund E, Sorlie PD, & Loveless CA (2000). Marital status and mortality: The National Longitudinal Mortality Study. Ann Epidemiol, 2(10), 24–38. 10.1016/s1047-2797(99)00052-6 10854957

[R24] Karl K, Peluchette J, & Schlaegel C (2010). Who’s posting Facebook faux pas? A cross‐cultural examination of personality differences. International Journal of Selection and Assessment, 18(2), 174–186.

[R25] Kopera AA, Maier RA, & Johnson JE (1971). Perception of physical attractiveness: The influence of group interaction and group coaction on ratings of women. Proceedings of the Annual Convention of the American Psychological Association.

[R26] Lee DS, Jiang T, Crocker J, & Way BM (2022). Social media use and its link to physical health indicators. Cyberpsychology, Behavior, and Social Networking, 25(2), 87–93. 35021894 10.1089/cyber.2021.0188 PMC8864418

[R27] Leschak CJ, & Eisenberger NI (2019). Two distinct immune pathways linking social relationships with health: inflammatory and antiviral processes. Psychosomatic medicine, 81(8), 711. 31600173 10.1097/PSY.0000000000000685 PMC7025456

[R28] Lima ML, Marques S, Muiños G, & Camilo C (2017). All you need is Facebook friends? Associations between online and face-to-face friendships and health. Frontiers in Psychology, 8, 68. 28194125 10.3389/fpsyg.2017.00068 PMC5278495

[R29] McElhaney KB, & Allen JP (2001). Autonomy and adolescent social functioning: The moderating effect of risk. Child Development, 72(1), 220–235. 11280481 10.1111/1467-8624.00275 PMC1551976

[R30] Merz EL, Roesch SC, Malcarne VL, Penedo FJ, Talavera GA, Castañeda SF, & Gallo LC (2016). Social support, simpatía, and hypertension prevalence in Hispanics/Latinos: Findings from the HCHS/SOL Sociocultural Ancillary Study. Journal of Latina/o Psychology, 4(3), 131. 28804695 10.1037/lat0000047 PMC5553902

[R31] Mikami AY, Szwedo DE, Allen JP, Evans MA, & Hare AL (2010). Adolescent peer relationships and behavior problems predict young adults’ communication on social networking websites. Developmental Psychology, 46, 46–56. 20053005 10.1037/a0017420 PMC2855302

[R32] Mikami AY, Szwedo DE, Ahmad SI, Samuels AS, & Hinshaw SP (2015). Online social communication patterns among emerging adult women with histories of childhood attention-deficit/hyperactivity disorder. Journal of Abnormal Psychology, 124, 576–588. 25894439 10.1037/abn0000053 PMC4573788

[R33] Mikami AY, & Szwedo DE (2011). Social networking in online and offline contexts. Encyclopedia of Adolescence, 2801–2808.

[R34] Miller RE (2020). College students and inappropriate social media posting: Is it a question of personality or the influence of friends? Personality and Individual Differences, 158, 109857.

[R35] Mrug S, Molina BS, Hoza B, Gerdes AC, Hinshaw SP, Hechtman L, & Arnold LE (2012). Peer rejection and friendships in children with attention-deficit/hyperactivity disorder: Contributions to long-term outcomes. Journal of abnormal child psychology, 40, 1013–1026. 22331455 10.1007/s10802-012-9610-2 PMC3384771

[R36] Nabi RL, Prestin A, & So J (2013). Facebook friends with (health) benefits? Exploring social network site use and perceptions of social support, stress, and well-being. Cyberpsychology, Behavior, and Social Networking, 16(10), 721–727. 23790356 10.1089/cyber.2012.0521

[R37] Narr RK, Allen JP, Tan JS, & Loeb EL (2019). Close Friendship Strength and Broader Peer Group Desirability as Differential Predictors of Adult Mental Health. Child Development, 90(1), 298–313. 28832975 10.1111/cdev.12905 PMC5821600

[R38] Nesi J, Choukas-Bradley S, & Prinstein MJ (2018b). Transformation of adolescent peer relations in the social media context: Part 2—application to peer group processes and future directions for research. Clinical child and family psychology review, 21(3), 295–319. 29627906 10.1007/s10567-018-0262-9 PMC6402323

[R39] Nesi J, Choukas-Bradley S, & Prinstein MJ (2018a). Transformation of adolescent peer relations in the social media context: Part 1—A theoretical framework and application to dyadic peer relationships. Clinical child and family psychology review, 21(3), 267–294. 29627907 10.1007/s10567-018-0261-x PMC6435354

[R40] Patzer GL (1985). Measurement of Physical Attractiveness. The Physical Attractiveness Phenomena, 15–41.

[R41] Politte‐Corn M, Nick EA, & Kujawa A (2023). Age‐related differences in social media use, online social support, and depressive symptoms in adolescents and emerging adults. Child and Adolescent Mental Health. 10.1111/camh.12640 PMC12051161 36751140

[R42] Poulin F, Dishion TJ, & Haas E (1999). The peer influence paradox: Friendship quality and deviancy training within male adolescent friendships. Merrill-Palmer Quarterly, 42–61.

[R43] Resnik F, & Bellmore A (2019). Connecting online and offline social skills to adolescents’ peer victimization and psychological adjustment. Journal of youth and adolescence, 48, 386–398. 30390194 10.1007/s10964-018-0953-z

[R44] Riggio RE, Widaman KF, Tucker JS, & Salinas C (1991). Beauty is more than skin deep: Components of attractiveness. Basic and applied social psychology, 12(4), 423–439.

[R45] Roche TM, Jenkins DD, Aguerrevere LE, Kietlinski RL, & Prichard EA (2015). College Students’ Perceptions of Inappropriate and Appropriate Facebook Disclosures. Psi Chi Journal of Psychological Research, 20(2), 86–96.

[R46] Roulin N (2014). The influence of employers’ use of social networking websites in selection, online self‐promotion, and personality on the likelihood of faux pas postings. International Journal of Selection and Assessment, 22(1), 80–87.

[R47] Shakya HB, & Christakis NA (2017). Association of Facebook use with compromised well- being: A longitudinal study. American Journal of Epidemiology, 185(3), 203–211. 28093386 10.1093/aje/kww189

[R48] Shrout PE, & Fleiss JL (1979). Intraclass correlations: Uses in assessing rater reliability. Psychological Bulletin, 86, 420–428. 18839484 10.1037//0033-2909.86.2.420

[R49] Slavich GM (2020). Social safety theory: a biologically based evolutionary perspective on life stress, health, and behavior. Annual Review of Clinical Psychology, 16, 265–295. 10.1146/annurev-clinpsy-032816-045159 PMC7213777 32141764

[R50] Szwedo DE, Mikami AY, & Allen JP (2012). Social networking site use predicts changes in young adults’ psychological adjustment. Journal of Research on Adolescence, 22(3), 453–466. 23109797 10.1111/j.1532-7795.2012.00788.x PMC3482112

[R51] Uchino BN, de Grey RGK, Cronan S, Smith TW, Diener E, Joel S, & Bosch J (2018). Life satisfaction and inflammation in couples: An actor–partner analysis. Journal of Behavioral Medicine, 41, 22–30. 28884245 10.1007/s10865-017-9880-9 PMC5766426

[R52] Ware JE, & Sherbourne CD (1992). The MOS 36-item short-form health survey (SF-36): I. Conceptual framework and item selection. Medical Care, 30(6), 473–483. 1593914

[R53] Wolfer L (2017). No sex, cursing and politics: Adult views of inappropriate Facebook posts. Journal of Human Values, 23(2), 116–128.

